# 

**Published:** 1888-06-30

**Authors:** 


### Special Notice to Newsagents, Advertisers, and the Trade.

**CHANGE OF OFFICE AND PUBLISHER.**

Notice is hereby given that the Office of The Hospital will henceforth be at 38, Old Jewry (Cheapside end), E.C., to which address all communications should be sent. Mr. A. Doig has been appointed Advertising Agent, and Mr. J. H. S. Hanning, Manager. All Cheques and Post Office Orders should be made payable to J. H. S. Hanning, 38, Old Jewry, E.C., crossed Lloyds, Barnetts, and Bosanquets Bank (Limited).

### To Secretaries and Matrons.

The Editor will be glad to have a copy of the Regulations, and also of every Report issued from the Press of Asylums, Hospitals, Convalescent and Nursing Institutions, as well as those of other Charities, so soon as they are published, and an early copy in duplicate of all appeals for funds. Notices of Annual and other Meetings, Festival Dinners, etc., will be welcomed. All such communications should be addressed to *The Editor, The Lodge, Porchester Square, London, W.* Any superintendent, secretary, matron, or other chief official who will regularly send such documents and information will be registered to receive free of cost a cover for binding The Hospital in half-yearly volumes on sending name and address to the publisher.

### AMUSEMENTS WITH PROFIT FOR ALL AGES.

**Rules.**—All answers must be addressed to the PRIZE EDITOR, THE HOSPITAL, 38, Old Jewry, Cheapside, London, E.C., not later than the *first* post on Thursday next. The full name and address must in all cases be given, but a *nom de plume* may be added for publication. Only one side of the paper must be written on.

### FOR MEN AND WOMEN.

### First Quarterly Poetical Competition.

Commenced May 26th, 1888; ends August 25th, 1888.

This quarter a PRIZE of ONE GUINEA will be given to the competitor who gains the highest number of marks for original poems, on any subjects suitable for insertion in this paper. All contributions sent in will be credited according to merit, eighteen marks being the highest score for any one contribution. This competition to alternate weekly with the set acrostic competition.

### Acrostic Competition.

This quarter the original acrostic competition will be discontinued, and A PRIZE of HALF-A-GUINEA will be given to the competitor who gains the highest score for solution of acrostics set. *One mark* only will be given to the competitors who solve all the lights of each acrostic.

Exception will be made in the case of *quotation acrostics*, when *two marks* will be given, *one* for the correct solution of all *lights*, and *one* mark for the *source* of the quotations.

This week credit will be given for

**No 3. Original Poem.**

### Results of No. 2. Original Poem

Dodo (15), K.M. (15), Hesperornis (14), Patience (13), A.M.E (13), S.M.W., (10).

### On the Death of the late Emperor Frederick of Germany.

In the land of our fathers the people are wailing
An Emperor noble, courageous, and true,
Their sorrow how touching yet all unavailing;
For Germany's Sovereign has paid Nature's due.

In the Royal procession to Westminster Abbey,
On the Jubilee day, with its splendour and pride;
C, who 'mong the princes less careworn and crabby,
Than *he* in white uniform—sword at his side?

Struck down by a malady strange and insidious,
That baffled the powers of all surgical skill;
His life ebbed and flowed, till nature perfidious,
Declared on a day that death was her will.

The tragedy solemn at length has been ended,
The Emperor Frederick at last has gone home;
The knees of the nation "en masse" are now bended,
Their hearts throb with grief, as they think of his tomb.

God grant the new Emperor, William the Second,
Strength from on high, and wisdom as dew;
That he, like his ancestors, too, may be reckoned,
As noble, courageous, yet tender and true.

HESPERORNIS.

### THE WORD COMPETITION.

Third Quarterly Competition commenced

May 5th, 1888; ends August 4th, 1888.

Three prizes of one guinea, 15*s*. and 10*s*. 6*d*. will be given each quarter to the three competitors who obtain the highest number of marks by making the largest number of words derived from the word or phrase set for anatomical dissection; that for this, the *eighth* week of the quarter, being

"MOONSHINE."

OBSERVE.—Proper names, abbreviations, foreign words, words of less than four letters, and repetitions are barred; plurals, and past and present participles of verbs are allowed. Nuttall's *Standard* dictionary only to be used.

N.B.—All words sent in must be arranged *alphabetically*, with correct total affixed.

### Results of Sixth Week.

*The Argosy*.—Patience (247), Tinie (241), Lightowlers (225), Lauriston (225), Sym (224), Dollie (216), Reldas (211), Dodo (193), Carmen (193), Oliver (189), Puff (187), Alicia (163).

### THE CHILDREN'S CORNER.

PRIZES.

FOR MOST CORRECT ANSWERS TO PUZZLES.

This Commenced October 1st, 1887.

One Pound a Month.

A PRIZE OF THREE GUINEAS

to the Competitor who shall have sent in the largest number of correct or best answers during the twelve months.

### Puzzles.—Fourth Week.

No. 15. ARITHMETICAL PUZZLE.—In a certain year of the *eighteenth* century a man said that 18 years earlier the square of his age gave the number of the year. What was his age when he said this?

No. 6. GEOGRAPHICAL ACROSTIC.—1. A town in Nottinghamshire. 2. A river in South America. 3. A city in Kent. 4. A country in Southern Hindoostan. 5. A favourite watering town in Sussex. 6. An English mountain. 7. A province of Spain. 8. Two vowels. 9. A city in Normandy. Initials give the name of an English town; the finals that for which it is noted.

No. 17. SQUARE WORD.

. . . . 1. Crippled.
. . . . 2. Sour, sharp.
. . . . 3. Possessive pronoun.
. . . . 4. Man's first abode.

No 18. CHARADE.

My first *dwells* in the mighty deep,
And sports in harmless play;
Oft chalky rocks my *second* keep
Hid from the light of day.
My *whole* within my first is found,
And now this riddle please expound.

### Results of Second Week Puzzles.

No. 6.—DOUBLE ACROSTIC.

W ainflee T
O dess A
L eitri M
F errarr A
E xete R

No. 7.—DECAPITATION.—Mouse, Ouse, Use.

No. 8.—FLORAL ANAGRAMS.—1, Chrysanthemum; 2, Neapolitan Violet 3, China Aster.

No. 9.—MISSING LETTER PUZZLE.

Under the greenwood tree,
Who loves to lie with me,
And tune his merry note
Unto the sweet bird's throat,
Come hither, come hither, come hither;
Here shall he see
No enemy,
But winter and rough weather.

—*As You Like It.*

No 10.—ARITHMETICAL PUZZLE.—160 oz. is the smallest mass from which an exact number of sovereigns could be coined.

As 17*s* 10½*d*. is $\frac{143}{160}$ of £1, it follows that £$3\frac{143}{160}$ weigh 1 oz.; ∴ 160 times £$3\frac{143}{160}$, or £623 might be coined from a mass of 160 oz., the weight required.

*All right.*—A. C. K., Scrooge. *Four right.*—Gem, Thanet, Pickles, Jumbo, Kensington. *Two right.*—Alsie, Plummy Fluff, R. H., Hercules.

### Conditions.

I. Every paper sent in must be clearly written, and one side only must be used. All competitors must mark at the head of their papers the number of their competition.

II. Each paper must be signed by the author with his or her real name and address. A *nom de plume* may be added, if the writer does not desire to be referred to by us by his real name. In the case of all prizewinners, however, the real name and address will be published.

III. All communications relating to prize competitions should be addressed to "The Prize Editor, THE HOSPITAL, 38, Old Jewry, London, E.C." Competitors are requested not to include in letters, etc., to the Prize Editor communications on matters of other business. All letters must be fully prepaid.

IV. The Prize Editor of THE HOSPITAL is the sole judge of the competitions, and his decision is in all cases final and without appeal.

V. The Prize Editor reserves the right to publish any paper sent in, whether it receives a prize or not. He does not hold himself responsible for any MSS. sent, nor can he undertake to return rejected competitions.

VI.—All children resident at school are requested to forward their *home* address in addition to the school one, when entering for the "Children's Corner" Competitions.

**Notice to all Correspondents.**—N.B.—All letters referring to this page, which do not arrive at 38, Old Jewry, Cheapside, London, E.C., by the *first post on Thursdays*, and are not addressed PRIZE EDITOR, will in future be disqualified and disregarded.

No one will be permitted to win the Monthly Prizes twice in any one year, the Annual Prize being offered especially to prevent this.

